# Determination of the Impact of High-Intensity Pulsed Electromagnetic Fields on the Release of Damage-Associated Molecular Pattern Molecules

**DOI:** 10.3390/ijms241914607

**Published:** 2023-09-27

**Authors:** Matej Kranjc, Tamara Polajžer, Vitalij Novickij, Damijan Miklavčič

**Affiliations:** 1 Faculty of Electrical Engineering, University of Ljubljana, Trzaska cesta 25, 1000 Ljubljana, Slovenia; matej.kranjc@fe.uni-lj.si (M.K.); tamara.polajzer@fe.uni-lj.si (T.P.); 2 Institute of High Magnetic Fields, Faculty of Electronics, Vilnius Gediminas Technical University, Plytinės g. 27, 10105 Vilnius, Lithuania; vitalij.novickij@vilniustech.lt; 3 Department of Immunology, State Research Institute Centre for Innovative Medicine, Santariskiu g. 5, 08410 Vilnius, Lithuania

**Keywords:** electromagnetic field, HI-PEMF, immune response, DAMP, ATP, calreticulin, HMGB1

## Abstract

High-Intensity Pulsed Electromagnetic Fields (HI-PEMF) treatment is an emerging noninvasive and contactless alternative to conventional electroporation, since the electric field inside the tissue is induced remotely by an externally applied pulsed magnetic field. Recently, HI-PEMF has been successfully used in the transfer of plasmid DNA and siRNA in vivo, with no or minimal infiltration of immune cells. In addition to gene electrotransfer, treatment with HI-PEMF has also shown potential for electrochemotherapy, where activation of the immune response contributes to the treatment outcome. The immune response can be triggered by immunogenic cell death that is characterized by the release of damage-associated molecular patterns (DAMPs) from damaged or/and dying cells. In this study, the release of the best-known DAMP molecules, i.e., adenosine triphosphate (ATP), calreticulin and high mobility group box 1 protein (HMBG1), after HI-PEMF treatment was investigated in vitro on three different cell lines of different tissue origin and compared with conventional electroporation treatment parameters. We have shown that HI-PEMF by itself does not cause the release of HMGB1 or calreticulin, whereas the release of ATP was detected immediately after HI-PEMF treatment. Our results indicate that HI-PEMF treatment causes no to minimal release of DAMP molecules, which results in minimal/limited activation of the immune response.

## 1. Introduction

The effects of magnetic fields have inspired research since Faraday’s experiments on magnetic induction between two coils in the early nineteenth century. Since then, the effects of electromagnetic fields on biological systems have been intensively studied for possible diagnostic and therapeutic use. Studies on the effects of externally applied electromagnetic fields on the cells have shown an influence on intracellular signal transduction pathways, cytoskeletal proteins involved in cell shape modification, changes in mitochondrial membrane potential and the production of reactive oxygen species (ROS) [1,2,3,4]. The ability of high-intensity pulsed electromagnetic fields (HI-PEMF) to increase cell membrane permeability has been reported in recent studies. Research has shown that HI-PEMF can affect both mammalian cells and microorganisms in vitro [5] as well as contactlessly induce molecular transmembrane transfer in small animals [6,7,8]. Different molecules have been used to demonstrate the effects of HI-PEMF on cell membrane permeabilization, including propidium iodide (PI) [9], YO-PRO-1 [10], lucifer yellow [11], cisplatin and bleomycin [6]. HI-PEMF has also been used to enhance cell death [5] and disrupt the blood–brain barrier [12]. Mechanisms and pathways of membrane permeability and thus consequently increased molecular transmembrane transport by the HI-PEMF are still not known. The effect is similar to the membrane permeabilization observed in conventional electroporation, a process that is triggered by high-intensity electric field pulses (hundreds of V/cm) of a short duration (µs–ms) [13]. One of the promising medical applications of conventional electroporation is gene electrotransfer (GET), a powerful method of DNA delivery for DNA vaccination and gene therapy [14,15]. Compared to DNA injection only, GET was shown to improve DNA entry into muscle cells, a most widely used target for GET, up to 100–1000 fold, including both the number of cells transfected and the level of DNA uptake [16,17,18,19,20]. In several reports, GET was described as an effective tool to elicit an immune response in small and large animal models [21,22,23], with numerous reports proving that this technique is effective in the stimulation of humoral and cellular immunity [24]. Initially, it was thought that the increase in antigen availability mediated by higher gene delivery was the only mechanism responsible for enhancement of the immune response to DNA vaccines. Nevertheless, recent data suggest that in addition to enhancing gene delivery, electroporation also provides adjuvant-like effects [25,26]. Several reports are available in which the local effect on the muscular tissue induced by electroporation alone, after or before DNA administration, was shown to be responsible for the generation of an inflammatory environment with immune cell infiltration [27]. Activation of the inflammatory response and immune system is desired in cancer therapies [28] and DNA vaccination, as it results in minor tissue damage that quickly resolves, and pro-inflammatory cytokines are released [27,29]. The immune response is however undesirable in gene therapy, e.g., monoclonal antibodies production where prolonged expression is desired, as it may eliminate transfected cells and affect the expression and secretion of transgenic proteins [30,31]. The immune response is triggered by immunogenic cell death (ICD), which is characterized by the release of damage-associated molecular patterns (DAMPs) from damaged or/and dying cells. Released DAMPs bind to pattern-recognition receptors (PRRs) of immune cells and elicit an immune response [32,33]. To date, the release of DAMP after increased permeabilization of the cell membrane has been studied exclusively after application of conventional electroporation using nanosecond [34,35,36,37], microsecond [37,38,39], or high-frequency irreversible electroporation (H-FIRE) pulses [28,37]. In these studies, DAMPs such as adenosine triphosphate (ATP), high mobility group box 1 protein (HMGB1) release and calreticulin externalization have been demonstrated. We have previously shown that the release of DAMPs and possibly the triggering of the immune response can at least in part be controlled by pulse parameters such as pulse duration and pulse type [37].

HI-PEMF application in GET is reported only in a few studies. In 2012, a magnetic nerve stimulator was applied for the permeabilization of cells in guinea pig skin in vivo to enhance uptake and expression of GFP plasmid DNA [8]. We later demonstrated that HI-PEMF can be applied for delivering siRNA molecules to silence enhanced green fluorescent protein (EGFP) in B16F10-EGFP mouse tumors in vivo [40]. Since siRNA delivery is a promising gene therapy approach for inactivating oncogenes and tumor suppressor genes involved in cancer disease [41], the results obtained demonstrate the potential use of HI-PEMF for cancer therapy. Recently, we also showed that HI-PEMF facilitates the delivery of large molecules of plasmid DNA (pEGFP-N1) in different tissues (muscle, skin and tumors). Interestingly, histological analysis of treated tissues showed that the introduction of plasmid DNA using HI-PEMF resulted in no tissue damage and significantly less infiltration of inflammatory mononuclear cells compared to GET using conventional electroporation [7]. Similarly, HI-PEMF did not elicit significant immune cell infiltration when applied in the electrotransfer of siRNA to silence enhanced green fluorescent protein in mice tumors [40]. Understanding the cell response to HI-PEMF is important for GET and also for other applications such as electrochemotherapy (ECT) [6], especially since the cell response can be triggered by pulses or therapeutic molecules alone, or can be a consequence of their synergistic effects. It was shown that not only ECT combined with the chemotherapeutic drug bleomycin can induce ICD, but that also ECT with other drugs (cisplatin and oxaliplatin) triggers ICD to a similar degree [42]. Electric pulses and chemotherapeutics alone do not always induce the release of DAMP (HMGB1 was released only in the presence of chemotherapeutics alone, whereas calreticulin was externalized after electric pulses alone) [43].

Therefore, we conducted a study to investigate the release of three different DAMP molecules (ATP, HMGB1 and calreticulin), known as the gold standard for predicting ICD, following HI-PEMF treatment of three different cell lines of different tissue origin (Chinese hamster ovary cells—CHO, mouse melanoma—B16F1, rat myoblasts—H9C2). We compared the results with the release of DAMP molecules by two different pulsed electric field (PEF) pulse protocols used in conventional GET. In addition, HI-PEMF was also compared to irreversible electroporation (IRE) where the release of DAMP was previously shown [37,38].

## 2. Results

### 2.1. Adenosine Triphosphate

**Figure 1:**
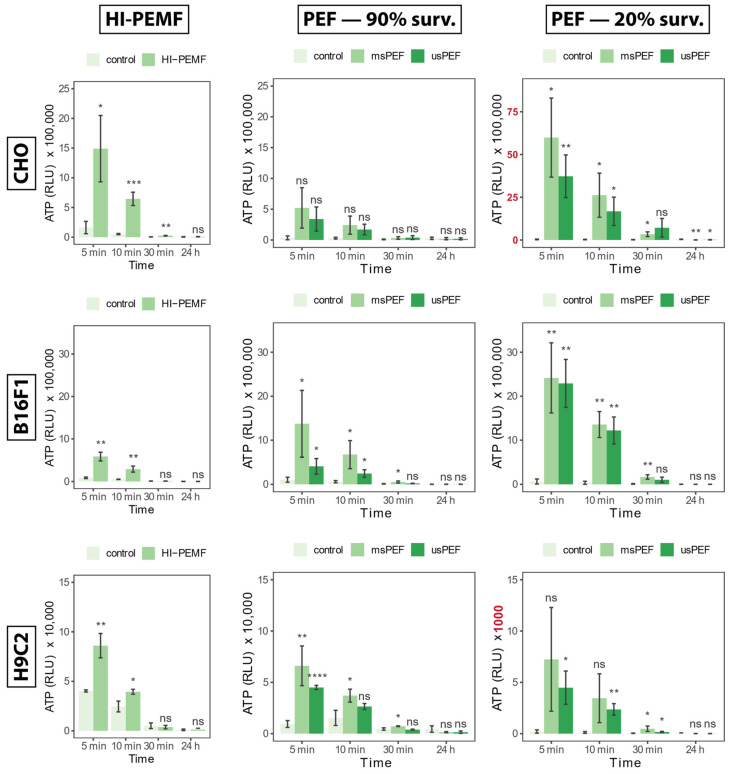
The release of ATP into the extracellular space 5, 10, 30 min and 24 h after HI-PEMF and PEF treatment in different cell lines. Asterisks *, **, ***, **** denote the statistical difference between the treated sample and the corresponding untreated control with a *p*-value of less than 0.05, 0.01, 0.005 and 0.001, respectively. Acronym “ns” denotes no statistical difference between the treated sample and the corresponding untreated control (*p* > 0.05). The mean ± standard deviation is given for each treatment. Note different scale ranges (colored red) in the release of ATP at PEF 20% survival in CHO and H9c2 cell lines.

Time points of ATP release into the extracellular space, measured for 24 h after treatment with HI-PEMF, are shown in Figure 1 for all three cell lines on the left side. A small increase in ATP can be observed in the untreated control as well, which is probably due to cell manipulation (e.g., pipetting). Compared to the untreated control, all HI-PEMF treated cells released significantly more ATP immediately after the treatment and with time, released ATP was decreasing. A similar trend was also observed after µsPEF and msPEF (Figure 1, middle, and right column). The signal of ATP release in HI-PEMF treatment is more similar to the ATP release during PEF treatment resulting in 90% survival, while ATP release in PEF treatment resulting in 20% survival is higher. However, no additional increase in ATP was observed within the 24 h after HI-PEMF or PEF treatment. Therefore, ATP release probably occurs due to changes in membrane permeability. Such dynamics of ATP release in HI-PEMF treatment is similar to PEF treatment [37].

### 2.2. Calreticulin Externalization

**Figure 2:**
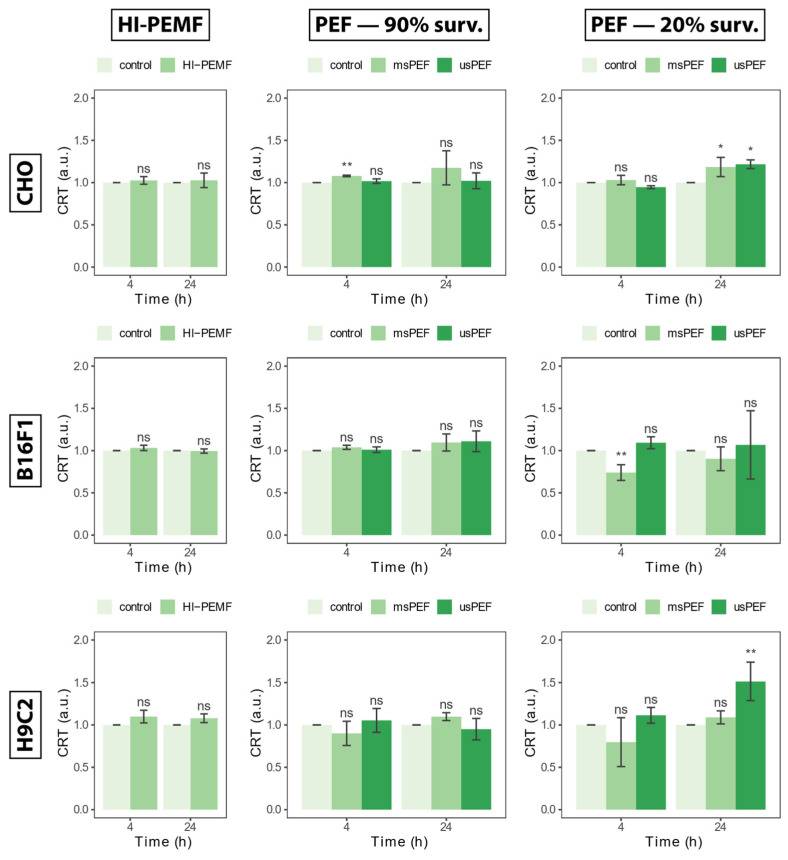
Externalization of calreticulin 4 and 24 h after HI-PEMF and PEF treatment on different cell lines. Asterisks *, ** denote the statistical difference between the treated sample and the corresponding untreated control with a *p*-value of less than 0.05 and 0.01, respectively. Acronym “ns” denotes no statistical difference between the treated sample and the corresponding untreated control (*p* > 0.05). The mean ± standard deviation is given for each treatment.

Transport of calreticulin to the outside of the cell membrane or externalization of calreticulin detected 4 and 24 h after HI-PEMF treatment is shown in Figure 2 on the left for all three cell lines. Compared to the untreated control, HI-PEMF-treated CHO, B16F1 and H9c2 did not cause any increased externalization of calreticulin. No statistical significance between treated and untreated samples was detected at 4 or 24 h after the treatment. A similar trend was also observed after µsPEF and msPEF treatment resulting in 90% survival (Figure 2, middle column). However, increased calreticulin can be observed after µsPEF and msPEF treatment resulting in 20% survival (Figure 2, right column), at least in CHO an H9c2. It seems that HI-PEMF treatment does not induce calreticulin externalization, similar to PEF treatments of low intensities (i.e., survival after treatment is around 90%).

### 2.3. High Mobility Group Box 1 Protein

**Figure 3:**
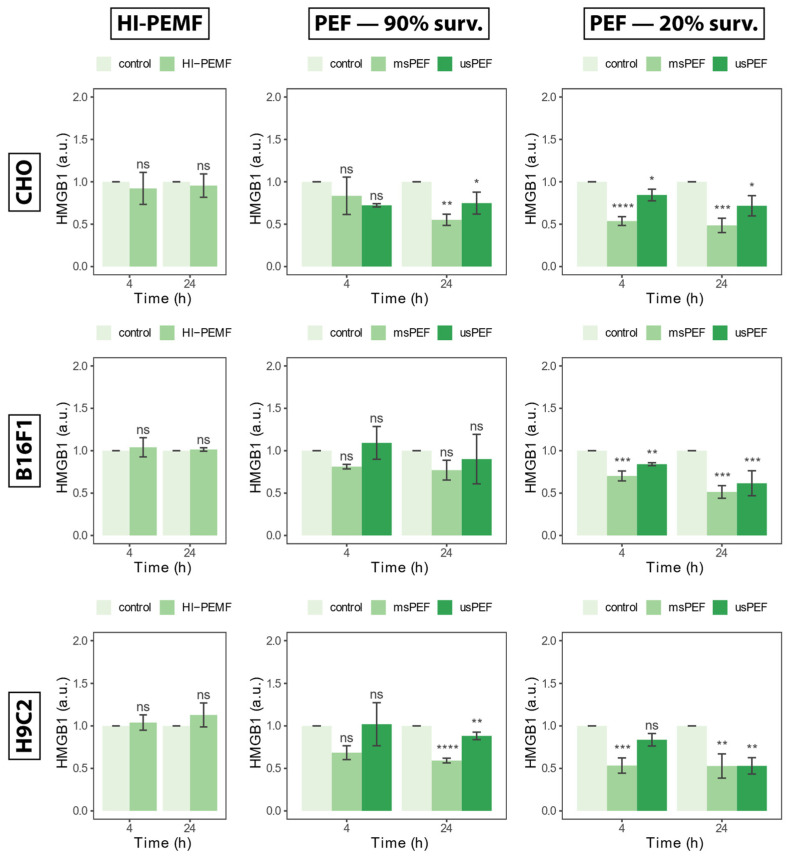
The release of HMGB1 in extracellular space 4 and 24 h after HI-PEMF and PEF treatment on different cell lines. Asterisks *, **, ***, **** denote the statistical difference between the treated sample and the corresponding untreated control with a *p*-value of less than 0.05, 0.01, 0.005, 0.001, respectively. Acronym “ns” denotes no statistical difference between the treated sample and the corresponding untreated control (*p* > 0.05). The mean ± standard deviation is given for each treatment.

The release of nucleic protein HMGB1 in the extracellular space measured 4 and 24 h after treatment with HI-PEMF, is shown in Figure 3 on the left for all three cell lines. Compared to the untreated control, treated CHO, B16F1 and H9c2 did not cause the release of HMGB1. No statistical significance between the treated and untreated sample was detected at 4 or 24 h after treatment. The absence of a statistical significance between the treated and untreated sample was also present in PEF treatment, resulting in 90% survival 4 h after treatment in all three cell lines (Figure 3, middle column). No statistically significant differences between control and PEF treatment resulting in 90% survival after 24 h were detected in B16F1, yet some differences were detected in the CHO and H9c2 cell line. Significant differences between the control and PEF treatment were detected in the PEF treatment resulting in 20% survival at 4 and 24 h after treatment. However, detected differences showed a lowered HMGB1 signal in the treated sample and it was not increased as would be expected in the presence of HMGB1 protein. In conclusion, no increase in the HMGB1 signal was observed in HI-PEMF- or PEF-treated cells, regardless of the cell line. This indicates that HI-PEMF treatment does not cause a release of nucleic protein HMGB1, the same as in PEF treatments of low intensities (i.e., survival after treatment is around 90%), which is consistent with previous results.

### 2.4. Temperature Increase Measurements

**Figure 4:**
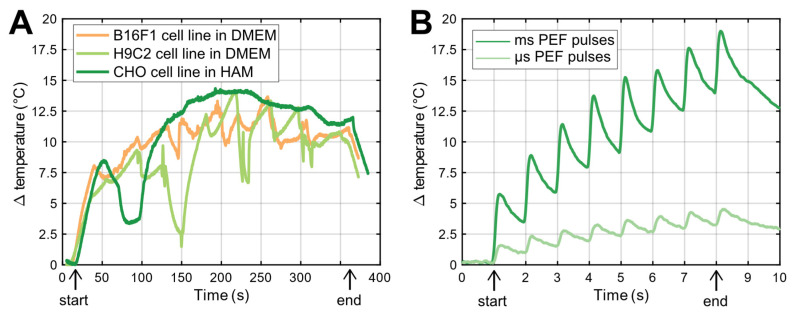
(**A**) Temperature change due to delivery of HI-PEMF pulses to three different cell suspensions. Deviation of temperature rise is due to repositioning and steering of ice (due to melting) in the ice bath where the applicator and PCR tube were placed during the delivery of HI-PEMF. The start and the end of pulse applications are marked as “start” and “end”, respectively. (**B**) Temperature changes due to delivery of µs- and msPEF pulses. Note different time scale in (**A**,**B**).

Figure 4 shows the temperature change of the cell suspensions during the delivery of HI-PEMF (Figure 4A) and the PEF treatment (Figure 4B). The start and end of the delivery are marked “start” and “end”, respectively, in Figure 4. Since a different number of pulses was delivered at the same repetition frequency of 1 Hz, namely 350 in the case of HI-PEMF and 8 in the case of PEF treatment, the suspensions were exposed to Joule heating for different durations, namely 350 s in the case of HI-PEMF and 8 s in the case of PEF treatment. The temperature change remained below 15 °C and 19 °C during the HI-PEMF and PEF treatment, respectively. The thermal effects on cells should be negligible, since the highest absolute temperature of the cell suspension during the application of HI- PEMF and both PEF treatments did not rise above the critical temperature of 43 °C, i.e., the threshold for thermal damage [44].

To evaluate the effect of temperature increase on the release of DAMP molecules, we performed an experiment in which we subjected cell suspensions to a similar temperature increase by placing them in a water bath at room temperature (25 °C) for the duration of HI-PEMF treatment (350 s), followed by an analysis of the release of DAMP molecules. We found no differences between the samples exposed to a temperature increase and control samples, i.e., samples exposed neither to a temperature increase nor to the application of pulses.

## 3. Discussion

In our study, we investigated the release of DAMP molecules (ATP, calreticulin and HMGB1), which are considered the gold standard for ICD, following HI-PEMF treatment of different cell lines (CHO, B16F1, H9C2). We compared the results with the release of DAMP molecules by two different PEF pulse protocols used in conventional electroporation with parameters used for gene electrotransfer (GET) and electrochemotherapy (ECT). In addition, HI-PEMF was also compared with IRE where the release of DAMP was previously shown.

Conventional electroporation is considered to be a universal method and a platform technology since all types of cells (animal, plant and microorganism) can be efficiently electroporated [45]. All electroporation applications require direct contact between the electrodes and the treated object, leading to a number of drawbacks, such as the dependence of electric field distribution on the dielectric properties of the sample [46,47,48,49], presence of electrochemical reactions in the electrode–electrolyte/tissue interfaces [50], changes in pH [51] and the possibility of electrical breakdown between the electrodes [52,53]. A direct comparison of conventional electroporation and electroporation induced by HI-PEMF shows that conventional electroporation is more effective than HI-PEMF, but the observed enhancement of molecule uptake is still substantial. Simplified calculations of the induced electric field show that the amplitudes obtained are 100–1000 times lower than those required for reversible electroporation in conventional electroporation using high-voltage pulses. Therefore, the mechanisms causing HI-PEMF mediated uptake are still not clear, although many have been proposed. The opening and closing of pores could be triggered by HI-PEMF induced hydrostatic pressure with membrane deformation and additional formation and accumulation of surface charges on the membrane due to magnetic force [54], lipid oxidation, electrophoresis [11], electroporation due to an induced electric field [6], altered receptor binding or activation [55] and mechanical stress induced by magnetic and electric fields [56,57]. One of the suggested mechanisms of HI-PEMF-mediated uptake of molecules is also electro-endocytosis [11,58,59], as it has been successfully used to enhance the uptake of molecules using electric fields with values similar to those induced by HI-PEMF [60,61,62].

It was shown that HI-PEMF, like PEF, can be successfully used for a GET in vitro and in vivo experimental setup. Furthermore, HI-PEMF can even be used for ECT. GET and ECT treatment performed in vivo usually result in activation of the immune response. Since GET and ECT treatments are a combination of therapeutic molecules and delivered pulses, both can activate the immune response. In our recent study, we showed that PEF treatment (which resulted in 90% survival) used in GET (ms pulses) and ECT (µs pulses) did not cause activation of the immune response in the absence of nucleic acids or chemotherapeutic drugs. No significant increase in HMGB1 or calreticulin externalization was observed with PEF treatment, which resulted in 90% survival, similar to the pulses used in ECT and GET. Only some ATP release was observed, which presumably occurred due to changes in permeability of the cell membrane [37]. Therefore, activation of the immune response in PEF may be triggered either by the presence of foreign nucleic acids or chemotherapeutic agents, or by the synergistic effect, i.e., the combined action of electrical pulses and foreign nucleic acids or chemotherapeutic agents. However, externalization of calreticulin was observed in PEF treatment that resulted in 20% survival, suggesting that PEF can also induce an immune response alone.

Whether the same applies for HI-PEMF treatment remains unknown. So far, only a weak activation of the immune system has been observed after GET treatment with HI-PEMF (detected by the low number of infiltrating immune cells). Our results show that HI-PEMF itself does not cause the release of HMGB1 or calreticulin, but only some ATP. ATP was released immediately after HI-PEMF treatment and afterwards started to decrease and remained low (same level as control) for the next 24 h. Biological cells deplete intracellular ATP or release it into extracellular space either under basal conditions or in response to stress or certain stimuli [63,64], which include oxidative and mechanical stimuli or membrane damage in the case of electroporation. Acute depletion or release of ATP during irreversible [65] and reversible electroporation [38,66,67] have been reported, indicating dose-dependent damage-associated molecular patterns following pulsed electric field treatment, which may have an effect on local inflammatory responses and possibilities for immunomodulation [68]. Therefore, we believe that ATP release in HI-PEMF is passive (i.e., driven by concentration gradient) and occurs due to a transient increase in cell membrane permeability. Our data on the effects of HI-PEMF show no controversy with studies using conventional electroporation, defining even more similarities between the two techniques.

On the one hand, the absence of an immune response would make GET treatment with HI-PEMF useful in gene therapies, where activation of the immune system reduces the chances of successful treatment. On the other hand, the absence of an immune response would make HI-PEMF treatment less efficient in DNA vaccination where an immune response is favorable. The immune response is also responsible for the success of ECT treatment in addition to increased toxicity and decreased blood flow. Since our previous study showed that HI-PEMF can be successfully used for ECT, it remains to be elucidated whether HI-PEMF can induce an immune response in combination with a chemotherapeutic agent.

## 4. Materials and Methods

### 4.1. Application of HI-PEMF and PEF

We applied HI-PEMF using a custom-made generator and an applicator that consisted of a round coil with 48 turns as described previously [9]. Briefly, the generator sent unipolar electric pulses to the applicator, which generated a time-varying magnetic field in the effective volume of the coil. The inner diameter of the coil was matched to the tip of a standard 0.2 mL sterile PCR tube where the cells were placed for treatment. The magnetic field was 6.7 T in the middle of the coil, and the induced electric field was up to 20 V/cm near the coil windings, declining to 0 at the center. We used the most efficient parameters from the previous study [9]. The survival (%) after HI-PEMF was 90 ± 3, 83 ± 10 and 91 ± 9 for CHO, B16F1 and H9C2, respectively. To mitigate Joule heating during pulse delivery, the HI-PEMF applicator was placed in an ice bath to cool it down.

**Figure 5:**
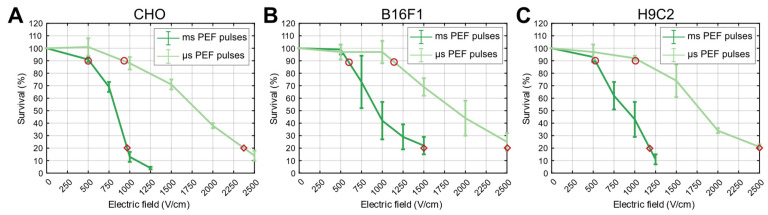
Survival curves obtained by applying µsPEF and msPEF treatment for CHO (**A**), B16F1 (**B**) and H9C2 (**C**) cell lines. Experimental points for DAMP analysis were determined based on survival curves, where the curves intersected at 90% (red circle) and 20% survival (red diamond).

For the application of the PEF treatment (µsPEF, msPEF), we applied electric pulses to cells in suspension in 2 mm electroporation cuvettes (VWR, Radnor, PA, USA) using the L-POR V0.1 electrical pulse generator (mPOR, Ljubljana, Slovenia) for µsPEF and the laboratory prototype pulse generator, previously described in [69] for msPEF. The pulse parameters for all protocols used in our study are listed in Table 1. The electric pulse parameters of the PEF treatments were chosen so that the cell survival rate (Figure 5) was similar to that of HI-PEMF (90%). We also performed an additional DAMP release analysis at a survival rate of 20%, which corresponds to irreversible electroporation.

The temperature rises due to the application of HI-PEMF and PEF pulses were monitored using a fiber optic sensor system (opSens, Québec, QC, Canada), which included a ProSens signal conditioner and an OTG-M170 fiber optic temperature sensor placed inside cell growth medium during the application of either HI-PEMF or PEF.

### 4.2. Cell Preparation

All cell lines were obtained from the European Collection of Authenticated Cell Culture. Chinese hamster ovary (CHO) cells were grown in HAM-F12 growth medium (PAA, Leonding, Austria), while mouse melanoma cells B16F1 and rat H9c2 hearth myoblasts were grown in DMEM growth medium (Sigma-Aldrich, St. Louis, MO, USA). All three growth media (500 mL) were supplemented with 50 mL of fetal bovine serum (Sigma-Aldrich, St. Louis, MO, USA), L-glutamine (2.5 mL for CHO, 5 mL for B16F1, 10 mL for H9c2) (StemCell, Vancouver, BC, Canada), 50 µL of penicillin/streptomycin (PAA, Leonding, Austria) and 500 µL of gentamycin (Sigma-Aldrich, St. Louis, MO, USA), i.e., complete growth media. Such media were used throughout the experiments. Cells were subcultured every 3–4 days and incubated at 37 °C in a humidified atmosphere with a 5% CO_2_ incubator. Passages numbered 5 to 25 were used in experiments. After reaching 70% confluency, cells were detached with trypsin solution (10× trypsin-EDTA, PAA, Leonding, Austria) in a ratio of 1:9 diluted in Hank’s basal salt solution (StemCell, Vancouver, BC, Canada). Trypsin was inactivated after 2–3 min by the growth medium. After centrifugation (5 min at 180× *g* and 22 °C), supernatant was removed and cells were resuspended in growth medium to a desired cell density. For µsPEF and msPEF, cell density at 1 × 10^6^ cells/mL was used, from which 150 µL was transferred to 2 mm cuvettes (VWR, Radnor, PA, USA). For HI-PEMF, 3.75 × 10^6^ cells/mL were used from which 40 µL were transferred to a 0.2 µL PCR tube (ABgene, Thermo Fisher Scientific, Waltham, MA, USA).

### 4.3. Viability Assay

After pulse application, samples were diluted in fresh complete growth media (every cell line in their own medium) to obtain 1.5 × 10^5^ cells/mL and 100 µL of sample was plated in triplicates in a 96-well plate (TPP, Trasadingen, Switzerland). Samples were then incubated for 24 h at 37 °C and 5% CO_2_. After 24 h, 20 µL of MTS tetrazolium compound (CellTiter 96 AQueous One Solution Cell Proliferation Assay, Promega, Madison, WI, USA) was added to the samples and incubated for an additional 2 h. Then, the absorbance of reduced MTS tetrazolium compound was measured with a multiplate reader (Tecan Infinite M200, Tecan, Grödig, Austria) at 490 nm. The percentage of viable cells was obtained by the normalization of sample absorbance to the absorbance of the control (0 V).

### 4.4. Adenosine Triphosphate Assay

After pulse application, samples were diluted in fresh complete growth media (every cell line in their own medium) to obtain 1.5 × 10^5^ cells/mL and 50 µL of the diluted sample was plated in a white 96-well plate (Greiner, Kremsmünster, Austria). To each sample we added as well 100 µL of fresh complete growth media and 50 µL of ATP reagent (RealTime GloTM Extracelular ATP Assay, Promega, Madison, WI, USA). The 96-well plate with samples was then transferred to the multiplate reader Tecan, where the signal of luminescence was measured every 5 min for 24 h with a constant temperature set at 37 °C.

### 4.5. Calreticulin Assay

After pulse application, samples were diluted in fresh complete growth media (every cell line in their own medium) to obtain 1.5 × 10^5^ cells/mL and 500 µL of the diluted sample was transferred to a 48-well plate (TPP, Switzerland) and incubated at 37 °C and 5% CO_2_ for 4 or 24 h. Afterwards, cells were harvested, washed twice (400× *g*, 5 min) with ice-cold PBS buffer (Merck, Rahway, NJ, USA; Sigma-Aldrich, St. Louis, MO, USA) with 10% FBS. A total of 50 µL of primary antibody Calreticulin Monoclonal Antibody (Invitrogen, Waltham, MA, USA) diluted 1:100 in PBS buffer with 3% FBS, added to samples and incubated for 30 min at 4 °C in the refrigerator. Cells were then washed twice with ice-cold PBS buffer with 3% FBS. A total of 50 µL of secondary antibody Goat anti-Mouse IgG, Alexa Fluor 405 (Invitrogen, Waltham, MA, USA) diluted 1:250 in PBS buffer with 3% FBS was added to the cells and incubated for an additional 20–30 min at 4 °C in the refrigerator. Cells were then washed twice with ice-cold PBS buffer with 3% FBS and diluted in 50 µL of PBS buffer with 3% FBS. Before analysis, 5 µL of propidium iodide (PI) was added to the samples and incubated in the dark at room temperature for 15 min. The presence of a calreticulin signal was measured with a flow cytometer (Attune NxT; Life Technologies, Carlsbad, CA, USA). A 488 blue laser with a 574/26 nm band-pass filter was used for the detection of PI, while 405 nm with a 440/50 nm band-pass filter violet laser was used for the detection of the calreticulin signal. To obtain only a signal from calreticulin on the external membrane of the cell, the calreticulin signal was analyzed only on viable cells (PI negative cells). Median fluorescence of calreticulin on viable cells was obtained from a fluorescence intensity histogram, determined as the median value of the measured signal.

### 4.6. High Mobility Group Box 1 Protein Immunoassay

After pulse application, samples were diluted in fresh complete growth media (every cell line in their own medium) to obtain 1.5 × 10^5^ cells/mL and 80 µL of diluted sample was then transferred to a white 96-well plate (Greiner, Kremsmünster, Austria) and incubated at 37 °C and 5% CO_2_ for 4 or 24 h. Afterwards, HMBG1 was detected with Lumit™ HMGB1 Human/Mouse Immunoassay (Promega, Madison, WI, USA) according to the manufacturer’s instruction. A total of 20 µL of antibody mixture was added to the samples, followed by 90-min incubation in the dark at room temperature. Then, 25 µL of detection reagent was added to the samples and after 3 min luminescence was measured with the multiplate reader Tecan.

### 4.7. Statistical Analysis

Significant differences among treatment and control groups were evaluated by the Welch Two Sample *t*-test for HI-PEMF treatments and by parametric analysis of variance (ANOVA) and Tukey multiple comparison for PEF treatments, both at a significance level of 95% (*p* < 0.05). Results are expressed as mean ± standard deviations of replications (*n* = 3). Statistical analysis was performed in RStudio 2023 (R. RStudio, PBC, Boston, MA, USA).

## 5. Conclusions

Immune response is a major contributor to the success of electroporation-based therapies. Understanding the potential contribution of the immune response in HI-PEMF treatment is important because it can help estimate the outcome of treatment. HI-PEMF itself causes changes in membrane permeability, has little effect on survival and induces little or no immune response. However, further studies are needed to evaluate the contribution of the foreign DNA or chemotherapeutic drug to the immune response during treatment with HI-PEMF.

## Figures and Tables

**Table 1:** Parameters of the applied pulse protocols, where HI-PEMF represents treatment with high-intensity pulsed electromagnetic fields, whereas µsPEF and msPEF represent conventional electroporation treatment with micro- and millisecond pulses, respectively. Electric fields for PEF treatment are listed for two survivals (90% and 20%) and three different cell lines CHO/B16F1/H9c2.

Name of the Treatment	Electric Field (V/cm)		Magnetic Field (T)	Duration of Pulses (µs)	Number of Pulses	Repetition Frequency (Hz)
HI-PEMF	≤20		6.7	20	350	1
µsPEF	90% survival	20% survival	/	100	8	1
	1000/1125/1000	2500/2375/2500				
msPEF	90% survival	20% survival	/	5000	8	1
	500/625/500	1000/1500/1125				

## Data Availability

Data are available from the corresponding author on request.
